# Tumor cells fail to present MHC-II–restricted epitopes derived from oncogenes to CD4^+^ T cells

**DOI:** 10.1172/jci.insight.165570

**Published:** 2023-01-24

**Authors:** Spencer E. Brightman, Martin S. Naradikian, Rukman R. Thota, Angelica Becker, Leslie Montero, Milad Bahmanof, Ashmitaa Logandha Ramamoorthy Premlal, Jason A. Greenbaum, Bjoern Peters, Ezra E.W. Cohen, Aaron M. Miller, Stephen P. Schoenberger

**Affiliations:** 1 Division of Developmental Immunology, La Jolla Institute for Immunology, La Jolla, California, USA.; 2 Biomedical Sciences Program, School of Medicine, UCSD, La Jolla, California, USA.; 3 Division of Hematology and Oncology, UCSD Moores Cancer Center, La Jolla, California, USA.; 4 Novartis, San Diego, California, USA.; 5 IconOVir Bio, San Diego, California, USA.; 6 Bioinformatics Core and; 7 Division of Vaccine Discovery, La Jolla Institute for Immunology, La Jolla, California, USA.; 8 Department of Medicine, UCSD, La Jolla, California, USA.

**Keywords:** Immunology, Oncology, Antigen presentation, Cancer immunotherapy, T cell receptor

## Abstract

CD4^+^ T cells play a critical role in antitumor immunity via recognition of peptide antigens presented on MHC class II (MHC-II). Although some solid cancers can be induced to express MHC-II, the extent to which this enables direct recognition by tumor-specific CD4^+^ T cells is unclear. We isolated and characterized T cell antigen receptors (TCRs) from naturally primed CD4^+^ T cells specific for 2 oncoproteins, HPV-16 E6 and the activating KRAS^G12V^ mutation, from patients with head and neck squamous cell carcinoma and pancreatic ductal adenocarcinoma, respectively, and determined their ability to recognize autologous or human leukocyte antigen–matched antigen-expressing tumor cells. We found in both cases that the TCRs were capable of recognizing peptide-loaded target cells expressing the relevant MHC-II or B cell antigen-presenting cells (APCs) when the antigens were endogenously expressed and directed to the endosomal pathway but failed to recognize tumor cells expressing the source protein even after induction of surface MHC-II expression by IFN-γ or transduction with CIITA. These results suggest that priming and functional recognition of both a nuclear (E6) and a membrane-associated (KRAS) oncoprotein are predominantly confined to crosspresenting APCs rather than via direct recognition of tumor cells induced to express MHC-II.

## Graphical abstract

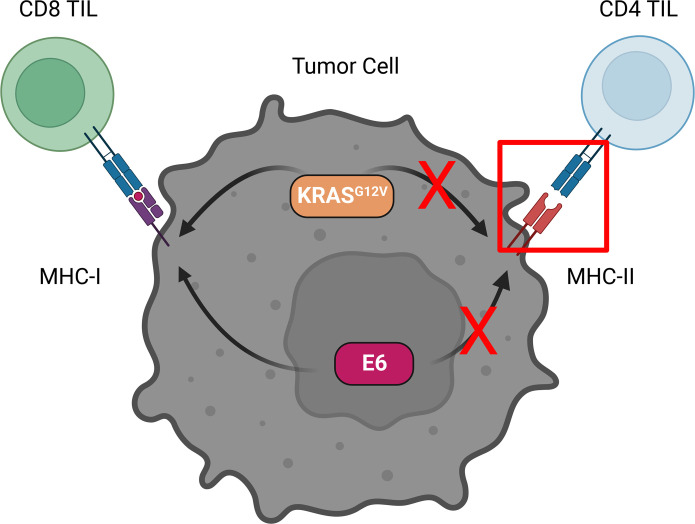

## Introduction

Malignant transformation of somatic cells is initiated by the action of activated oncogenes that dysregulate cell growth pathways and lead to unchecked proliferation (1). In the case of HPV-associated cancers, expression of the HPV early genes E6 and E7 contributes to oncogenesis by inhibiting the tumor suppressor genes p53 and Rb, respectively (2). Alternatively, constitutive oncogene activation may occur through somatic mutation in genes regulating cell growth and proliferation pathways, such as the RAS family. Indeed, activating KRAS mutations such as KRAS^G12V^ are commonly found in patients with pancreatic, colon, and lung cancers (3).

Although oncogenes promote disease, they can also provide targets for the host immune system. In recent years, the role of the immune system in controlling HPV-associated cancers has become widely recognized. CD8^+^ and CD4^+^ T cells recognizing HPV E6 and E7, as well as other HPV proteins, have been identified in both the peripheral blood and tumor-infiltrating lymphocytes (TILs) from patients with HPV-associated cancers (4–6). Immunotherapies targeting these antigens, including therapeutic cancer vaccines and adoptive cellular therapy (ACT) with TILs or TCR-engineered T cells, are an active area of preclinical and clinical investigation (2). T cell responses against oncogenic mutations have also been widely described (7–9). Furthermore, there is direct clinical evidence that ACT with TILs targeting mutant KRAS can promote objective responses (10).

Although CD8^+^ T cells recognizing epitopes derived from oncogenes mediate direct cytotoxicity against tumor cells (11–13), the role of CD4^+^ T cells in antitumor immunity is less well understood. Recent studies have highlighted a subset of CD4^+^ T cells expressing cytotoxic markers, such as granzyme B (GrzmB), in human cancers (14, 15). The antigen specificities of these cells, however, and thereby their therapeutic potential, remain largely unknown. Furthermore, for these cytotoxic CD4^+^ T cells to recognize and kill tumor cells, not only must the tumor cell express MHC-II, but also the relevant antigens must be directed to the appropriate presentation pathway.

In this study, we investigated the capacity of CD4^+^ T cells specific for the oncoproteins HPV-16 E6 and KRAS^G12V^ to recognize antigen-expressing tumor cells. Although both TCRs were able to directly recognize endogenously processed and presented antigen directed to the endosomal compartment of HLA-matched B cells, neither was capable of recognizing antigen-expressing tumor cells with induced expression of MHC-II. Overall, our results suggest that only certain antigens are presented directly on MHC-II^+^ tumor cells, which may limit the pool of CD4^+^ T cells capable of directly recognizing and eliminating tumor cells.

## Results

### Identification of HPV-16 E6-specific CD4^+^ and CD8^+^ T cell responses from TILs of head and neck squamous cell carcinoma.

**Figure 1:**
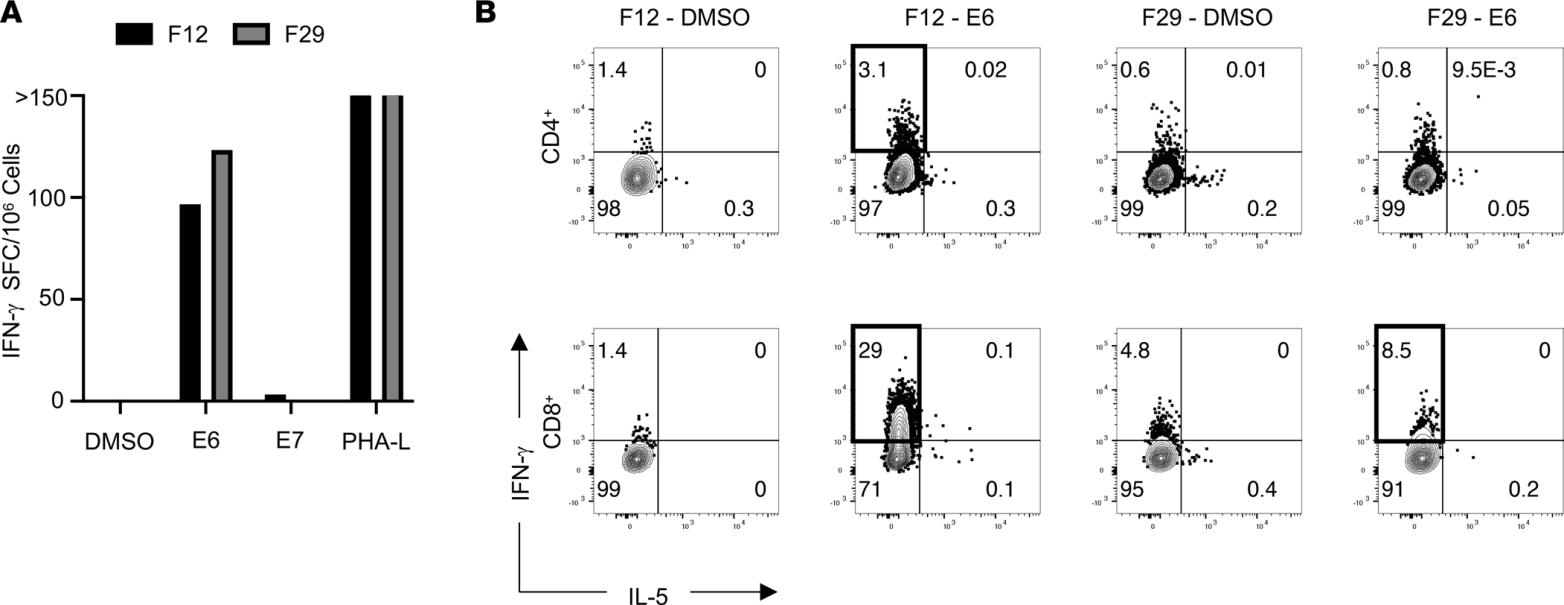
Identification of E6-reactive CD4^+^ and CD8^+^ TILs from HPV-16^+^ HNSCC. (**A**) IFN-γ ELISPOT assay results from TILs derived from different tumor fragment cultures stimulated with indicated peptide pools at 5 μg/mL or 5 μg/mL phytohemagglutinin-L (PHA-L) as a positive control. Data represent the average of 3 technical replicates in a single experiment. SFC, spot-forming cells. (**B**) Cytokine secretion assay results from TIL fragments with ELISPOT reactivity demonstrating IFN-γ and IL-5 production from both CD8^+^ and CD4^+^ T cell subsets after stimulation with 1 μg/mL of the indicated peptide pools. TIL subsets with E6-specific IFN-γ production greater than DMSO controls (designated with bolded gates) were sorted for TCR sequencing.

We screened a series of individual TIL cultures isolated from 2 to 4 mm tumor fragments from a patient (Hu-56) with HPV-16^+^ head and neck squamous cell carcinoma (HNSCC) (Supplemental Table 1; supplemental material available online with this article; https://doi.org/10.1172/jci.insight.165570DS1) by IFN-γ ELISPOT assays to identify T cell responses directed against HPV-16 viral oncoproteins E6 and E7, using peptide pools comprising overlapping 15-mers spanning the length of the E6 and E7 proteins. Notably, TIL fragments 12 and 29 generated a substantial number of spots over background when restimulated with the E6 peptide pool and contained both CD4^+^ and CD8^+^ T cells (Figure 1A and Supplemental Figure 1A). To identify the relevant T cell subsets, we analyzed these TIL cultures using a cytokine secretion assay. This revealed TIL fragment 29 (F29) contained E6-reactive CD8^+^ T cells, whereas fragment 12 (F12) contained both CD4^+^ and CD8^+^ T cells capable of recognizing the E6 peptide pool (Figure 1B). None of the CD4^+^ T cells produced IL-5 in response to E6 restimulation, validating a Th1-like phenotype.

To determine the TCR clonality of E6-specific TILs, we sorted single IFN-γ–secreting CD4^+^ T cells from F12 and CD8^+^ T cells from F12 and F29. TCR sequencing revealed a single TCR expressed by IFN-γ–secreting CD4^+^ T cells (*n* = 31 of 31 cells sequenced), as well as a single TCR clone shared by IFN-γ–secreting CD8^+^ T cells from both F12 and F29 (*n* = 40 of 41 and 37 of 38 cells sequenced, respectively) (Supplemental Table 2). These results indicate that monoclonal CD4^+^ and CD8^+^ T cells reactive to E6 are present within TILs from this patient.

### HLA-A*02:01–restricted CD8^+^ T cells from TILs recognize E6 presented by a patient-derived tumor cell line.

**Figure 2:**
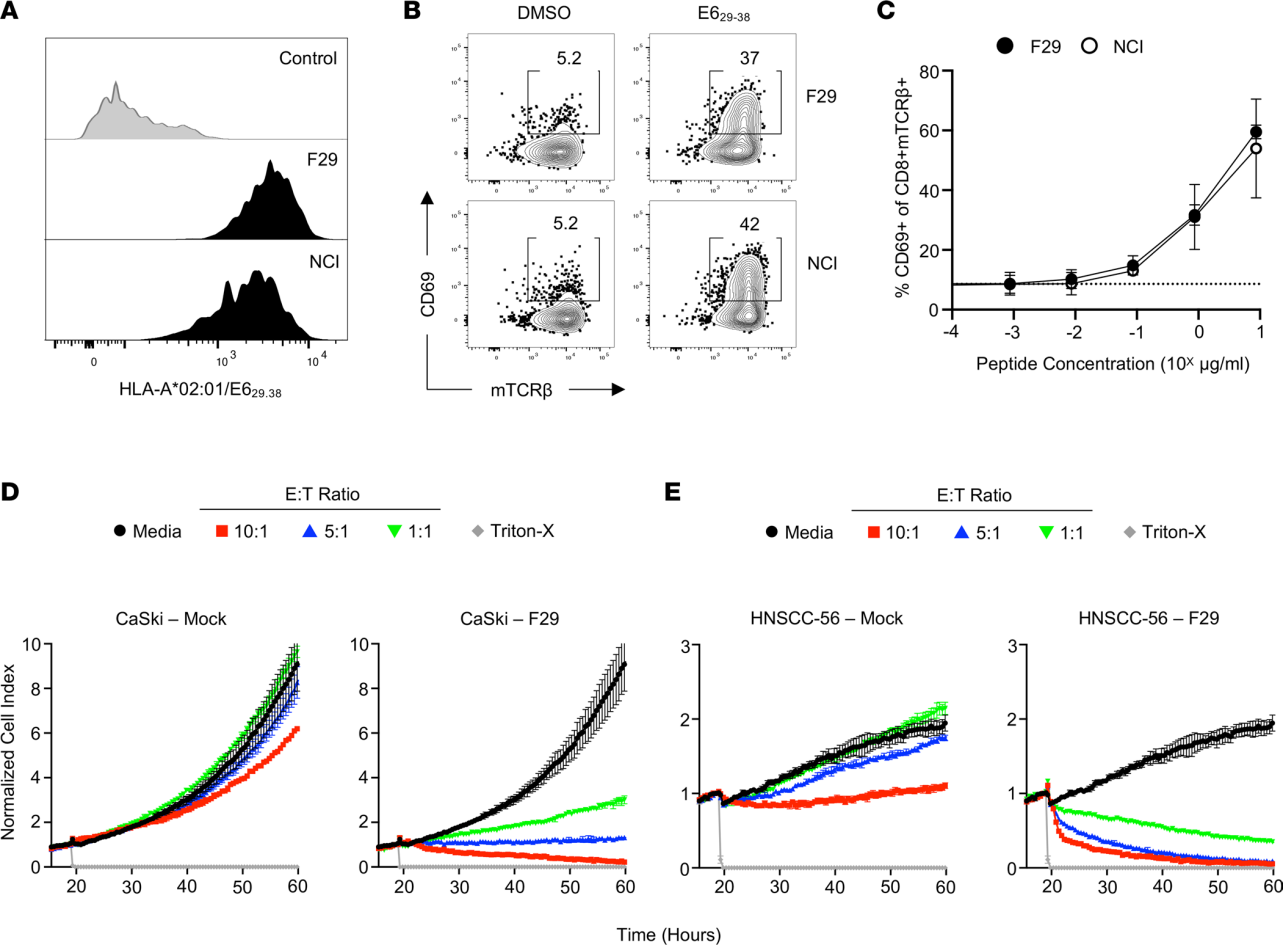
Functional characteristics of an HLA-A*02:01–restricted, E6-specific TCR from Hu-56 TILs. (**A**) Histogram demonstrating 1 μg/mL HLA-A*02:01/E6_29-38_ tetramer binding to Jurkat cells expressing an irrelevant TCR, a previously described TCR with known E6 specificity, or the TCR identified among the dominant CD8^+^ clonotype in patients’ TILs. (**B**) Representative FACS plots demonstrating specific upregulation of CD69 on Jurkat cells when stimulated with 1 μg/mL E6_29-38_ peptide. (**C**) Functional avidity curves comparing activation of TCR-expressing Jurkat cells stimulated with a titration of peptide concentrations. Data are presented as the mean ± SEM of 3 independent experiments. NCI, National Cancer Institute TCR. (**D**) Cellular impedance measurements to determine the kinetics of tumor cell death after recognition of CaSki cells by primary human T cells expressing the F29 TCR compared with mock-transduced donor T cells. Normalized cell index values over time for effector to target (E:T) ratios 10:1, 3:1, and 1:1 are shown compared with tumor cells cultured without effectors. Data are presented as mean values of 3 technical replicates ± SEM and are representative of 2 independent experiments. (**E**) Cellular impedance measurements to determine the kinetics of tumor cell death after recognition of an autologous HNSCC-56 tumor cell line by primary human T cells expressing the F29 TCR compared with mock-transduced donor T cells. Normalized cell index values over time for E:T ratios 10:1, 3:1, and 1:1 are shown compared with tumor cells cultured without effectors. Data are presented as mean values of 3 technical replicates ± SEM and are representative of 2 independent experiments.

TILs from F29 produced IFN-γ when restimulated with the E6_29-38_ peptide, suggesting that the F29 TCR recognized this previously described HLA-A*02:01–restricted epitope (Supplemental Figure 1B) (11). This was confirmed by tetramer-binding studies using E6_29-38_/HLA-A*02:01 to label TCR-deficient J76 cells expressing human CD8 and using a previously described E6_28-38_–specific TCR as a positive control (Figure 2A) (11). Both TCRs exhibited similar levels of functional avidity, as demonstrated by upregulation of the acute activation marker CD69 on J76 after stimulation with autologous B lymphoblastoid cell lines (B-LCLs) pulsed with titrated concentrations of E6_29-38_ peptide (Figure 2, B and C). These data validate the presence of a CD8^+^ T cell clone specific for a known epitope of E6 with similar functional characteristics to a TCR that has been tested in a clinical setting (16).

We next sought to determine whether the E6-specific CD8^+^ TCR could recognize endogenously expressed antigen and thereby mediate antitumor cytotoxicity. To do this, we used a well-characterized HLA-A*02:01^+^ HPV-16^+^ tumor line, CaSki, as well as an autologous tumor cell line, HNSCC-56, generated through cellular reprogramming of primary tumor tissue as previously described (17) for in vitro cytotoxicity assays. RNA-Seq analysis validated expression of HPV-16 early genes, including E6, by both the primary tumor sample and the autologous cell line (Supplemental Figure 1C). Primary human CD8^+^ T cells expressing the F29 TCR could mediate cell killing of both CaSki and HNSCC-56 at a range of effector-to-target ratios in vitro (Figure 2, D and E). Together, these data validate that the F29 TCR recognizes endogenously expressed E6 antigen.

### Identification of an HLA-DQ–restricted, E6-specific CD4^+^ T cell from TILs.

**Figure 3:**
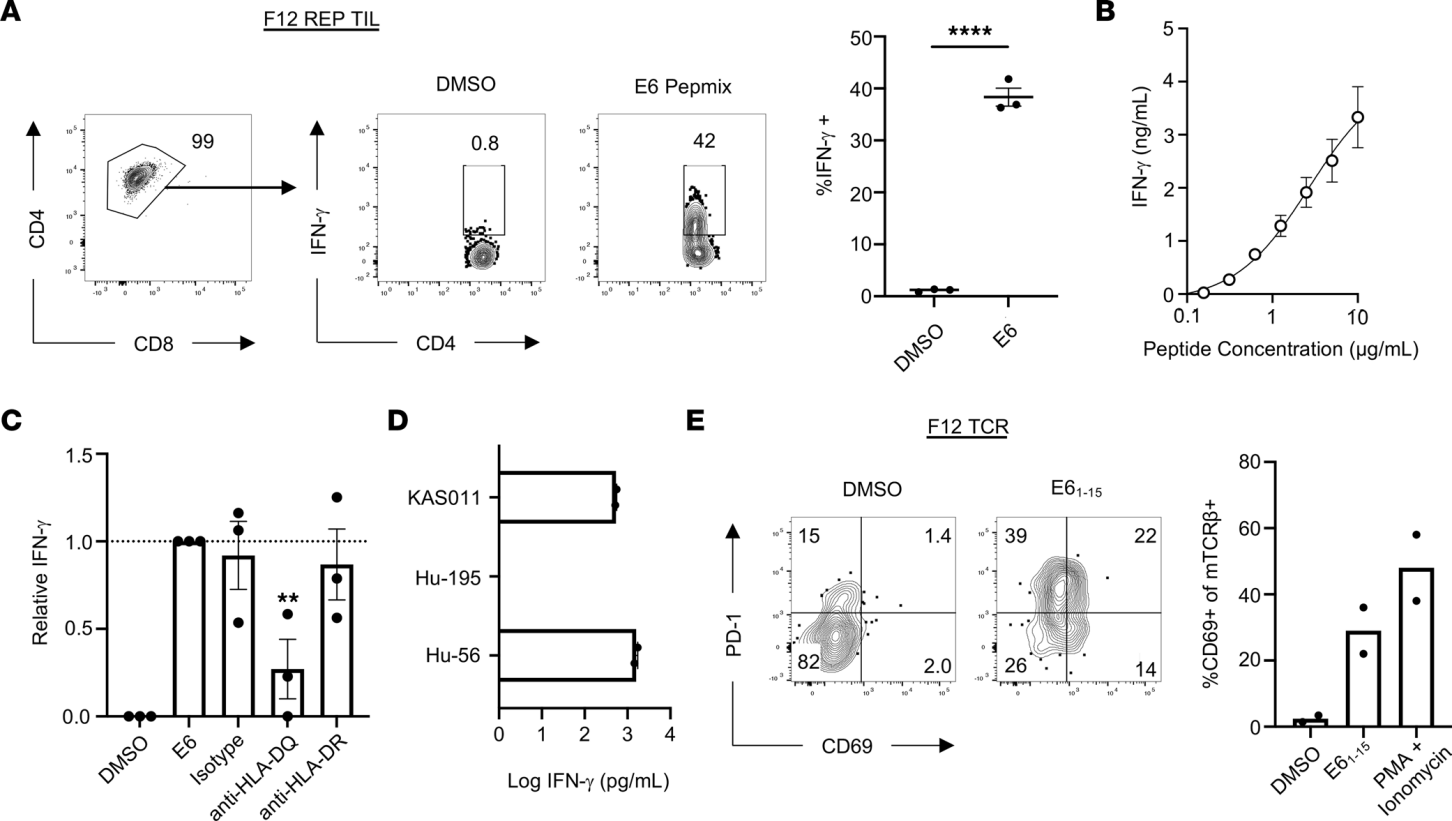
Functional characteristics of clonally expanded, HLA-DQ–restricted, E6-specific CD4^+^ TILs. (**A**) Representative FACS plots (left) and quantification (right) of intracellular cytokine staining for IFN-γ from TIL F12 after rapid expansion protocol after stimulation with 1 μg/mL E6 PepMix. Data represent the mean ± SEM of 3 independent experimental replicates. ****P < 0.0001, unpaired 2-tailed *t* test. (**B**) IFN-γ production by CD4^+^ TILs stimulated with a titration of E6_1-15_ peptide pulsed onto autologous B-LCLs. Data represent the mean ± SEM of 2 independent experimental replicates. (**C**) Normalized IFN-γ production by CD4^+^ TILs cultured with 1 μg/mL E6_1-15_ peptide-pulsed B-LCLs preincubated with indicated HLA-blocking Abs. Data represent the mean ± SEM of 3 independent experimental replicates. **P < 0.01, 2-way ANOVA. (**D**) IFN-γ production by CD4^+^ TILs stimulated with indicated B-LCLs pulsed with 1 μg/mL E6_1-15_ expressing mismatched HLA-DQ alleles. (**E**) Representative FACS plots (left) showing upregulation of activation markers PD-1 and CD69 after culturing TCR-transduced primary CD4^+^ T cells with autologous B-LCLs alone or pulsed with 1 μg/mL E6_1-15_. Quantification of CD69 expression by TCR-transduced primary CD4^+^ T cells (right). Data represent 2 independent experiments. mTCR-β, murine TCR-β.

To expand E6-specific TILs for further functional analysis, we used the previously described rapid expansion culture protocol with OKT-3 and irradiated allogeneic PBMCs to expand TILs from F12. Although the primary F12 culture originally contained both CD8^+^ and CD4^+^ T cells with E6 reactivity, the final cell product after rapid expansion contained 99% CD4^+^ T cells, of which approximately 38.33% demonstrated reactivity against E6, as demonstrated by intracellular cytokine staining for IFN-γ (Figure 3A). In addition to IFN-γ, stimulated E6-specific CD4^+^ TILs could secrete TNF-α but not IL-2 (Supplemental Figure 2A). Although the total frequency of GrzmB^+^ cells did not increase with antigen stimulation, gating on TNF-α–secreting cells revealed that E6-specific CD4^+^ TILs contained more stored intracellular GrzmB than did nonspecific TILs (Supplemental Figure 2B). IFN-γ–secreting CD4^+^ TILs also expressed the coinhibitory marker programmed cell death 1 (PD-1), consistent with published signatures of tumor-reactive CD4^+^ TILs (Supplemental Figure 2C) (18, 19). E6-specific CD4^+^ TILs demonstrated a high functional avidity, because these cells could secrete cytokines at peptide concentrations less than 1 μg/mL (Figure 3B).

To determine specificity of the F12 TIL culture, we pulsed autologous B-LCLs with individual peptides corresponding to each of the 37 peptides contained in the original E6 pool (Supplemental Table 3). CD4^+^ TILs from F12 secreted IFN-γ when stimulated with peptides E6_1-15_ and E6_5-19_, which share an 11–amino acid core sequence (Supplemental Figure 2D). Coculture experiments in the presence of HLA-blocking Abs revealed significantly diminished IFN-γ secretion by TILs when B-LCLs were blocked with anti–HLA-DQ Abs but not anti–HLA-DR or isotype control Abs (Figure 3C). To determine which HLA-DQ alleles are responsible for presentation of this epitope, we pulsed B-LCLs expressing known HLA-DQ alleles (Supplemental Table 4) with E6_1-15_ and found that KAS011 cells expressing HLA-DQA1*01:02 and DQB1*05:02, but not Hu-195 B-LCLs expressing DQA1*05:05 and DQB1*03:01, could present antigen to the TILs in a comparable manner to autologous Hu-56 B-LCLs (Figure 3D).

Finally, we sought to verify that the TCR sequence identified previously was indeed responsible for E6 recognition. Expression of the F12 TCR in primary human healthy donor CD4^+^ T cells was sufficient to promote recognition of the E6_1-15_ peptide, as evidenced by upregulation of CD69 and PD-1 (Figure 3E). Taken together, these results demonstrate the identification of an HLA-DQ–restricted epitope of E6 recognized by monoclonal CD4^+^ TILs.

### E6-specific CD4^+^ T cells do not recognize or kill autologous tumor cells expressing MHC-II.

**Figure 4:**
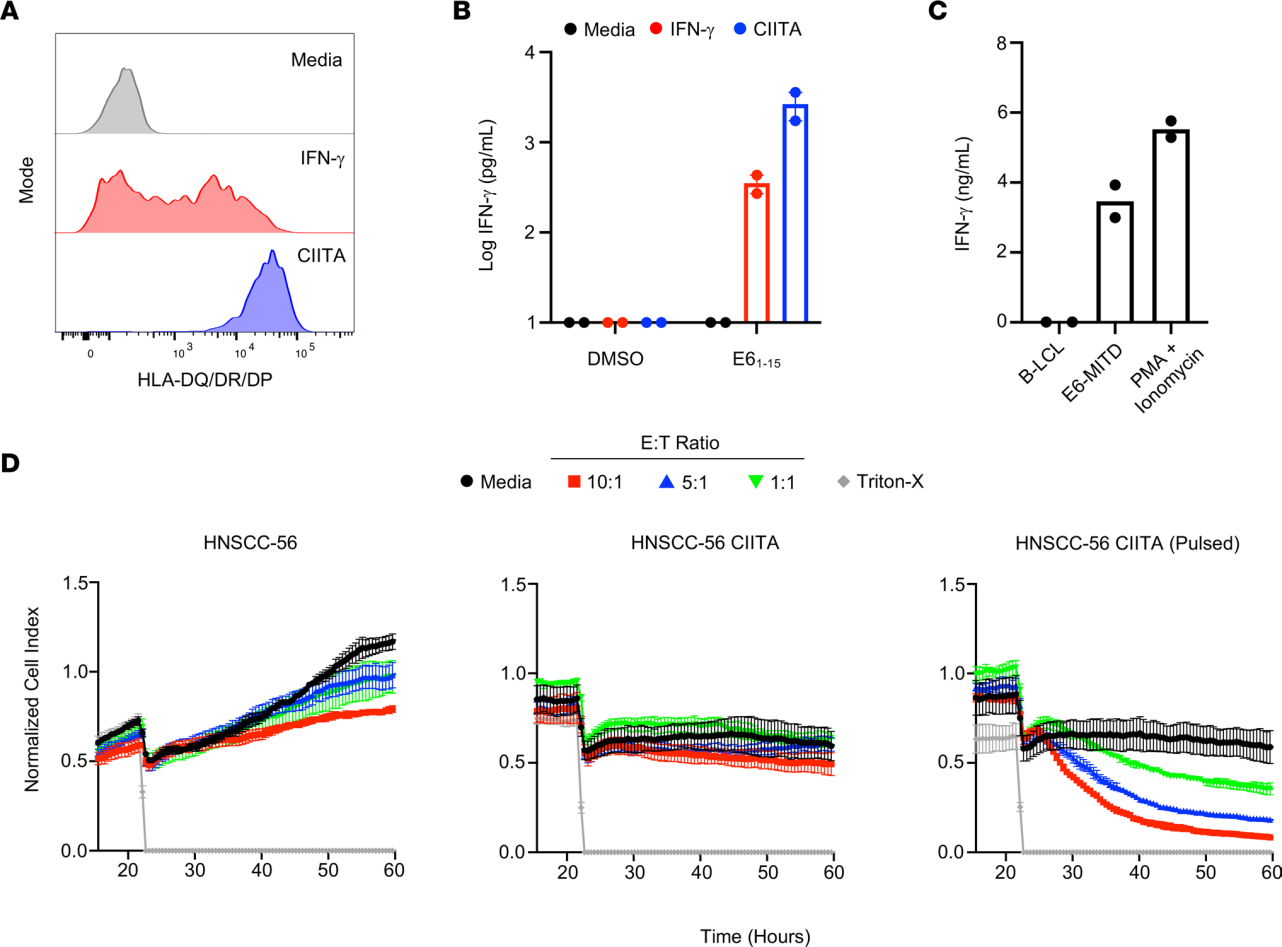
Autologous tumor cells do not present endogenous E6_1-15_ on MHC-II to CD4^+^ T cells. (**A**) Representative histograms of FACS staining for HLA-II surface expression on HNSCC-56 tumor cells left unstimulated, cultured with IFN-γ for 72 hours, or transduced with CIITA. (**B**) IFN-γ production by CD4^+^ TILs after culture with autologous tumor cells with or without addition of 1 μg/mL E6_1-15_ peptide. Data represent the mean ± SEM of 2 independent experimental replicates. (**C**) IFN-γ production by CD4^+^ TILs after culture with autologous B-LCLs, B-LCLs stably expressing E6_1-50_ with an endosomal targeting sequence, or PMA plus ionomycin. Data represent the mean of 2 independent experimental replicates. (**D**) Cellular impedance measurements to determine the kinetics of tumor cell death after recognition of an autologous HNSCC-56 tumor cell line by E6-specific CD4^+^ TILs compared with HNSCC-56 CIITA or HNSCC-56 CIITA pulsed with 1 μg/mL E6_1-15_. Normalized cell index values over time for effector to target (E:T) ratios 10:1, 3:1, and 1:1 are shown compared with tumor cells cultured without effectors. Data are presented as mean values of 3 technical replicates ± SEM and are representative of 2 independent experiments.

Recent studies have identified genetic signatures of cytotoxic CD4^+^ TILs in some human cancers (14, 15). However, whether cytotoxic CD4^+^ T cells play a role in HPV-driven cancers is unknown. To determine whether E6-specific CD4^+^ T cells from TILs could directly recognize HNSCC-56, we first investigated its MHC-II expression status. Although the tumor cells expressed no detectable surface MHC-II in culture, treatment with IFN-γ for 72 hours was sufficient to induce upregulation of MHC-II (Figure 4A).

Next, we cultured HNSCC-56 with CD4^+^ F12 TILs. Importantly, MHC-II^+^ tumor cells preconditioned with IFN-γ were unable to stimulate CD4^+^ TILs as measured by IFN-γ secretion (Figure 4B). However, IFN-γ–treated tumor cell lines pulsed with E6_1-15_ prior to the addition of TILs were able to stimulate a T cell response, indicating that surface expression of the restricting HLA-DQ alleles in a cell line that endogenously expresses E6 was not sufficient for tumor recognition but required exogenous loading of target peptide. The same requirement of exogenous target peptide loading was observed with CIITA-transduced tumor cells (HNSCC-56 CIITA), which constitutively express high levels of surface MHC-II (Figure 4B).

To test whether CD4^+^ TILs could recognize an endogenously processed and presented version of the E6 epitope on an APC, we retrovirally transduced autologous B-LCLs with an expression construct encoding the first 50 amino acids of E6 fused to the MHC-I transmembrane domain (E6-MITD), which induces localization of the fused amino acid sequence to the plasma membrane and supports presentation on MHC-II via endosomal trafficking (20). B-LCLs expressing the E6-MITD construct could stimulate E6-specific CD4^+^ TILs, indicating that the peptide epitope recognized by these TILs can be naturally processed and presented on MHC-II when directed to the appropriate pathway (Figure 4C).

Next, we sought to determine whether the F12 E6-specific CD4^+^ TILs were capable of direct tumor cytotoxicity. Consistent with the data from our recognition assays, E6-specific CD4^+^ TILs were unable to limit the growth of HNSCC-56 tumor cells, which do not express MHC-II (Figure 4D). Furthermore, HNSCC-56 CIITA tumor cells also grew uninhibited in the presence of CD4^+^ TILs. However, growth of HNSCC-56 CIITA tumor cells pulsed with E6_1-15_ peptide was inhibited in an effector cell dose-dependent manner. Overall, these data suggest that although HNSCC-56 can present E6 epitopes to CD8^+^ T cells, E6-specific CD4^+^ TILs are unable to directly recognize and lyse MHC-II^+^ autologous tumor cells.

Genes involved in antigen presentation are frequently mutated in cancers, which can prevent recognition by T cells (21). To determine whether HNSCC-56 tumor cells contained any somatic mutations precluding antigen presentation on MHC-II, we performed whole-exome sequencing of tumor cells and PBMCs from Hu-56 as a reference sample. Of the 104 identified nonsynonymous somatic mutations, there were no apparent mutations in components of the MHC-II presentation pathway, including *HLADMA*, *HLADMB*, and *CD74* (Supplemental Table 5).

### Identification of an HLA-DRB5*01:01–restricted, KRAS^G12V^-specific TCR from blood of a patient with pancreatic ductal adenocarcinoma.

**Figure 5:**
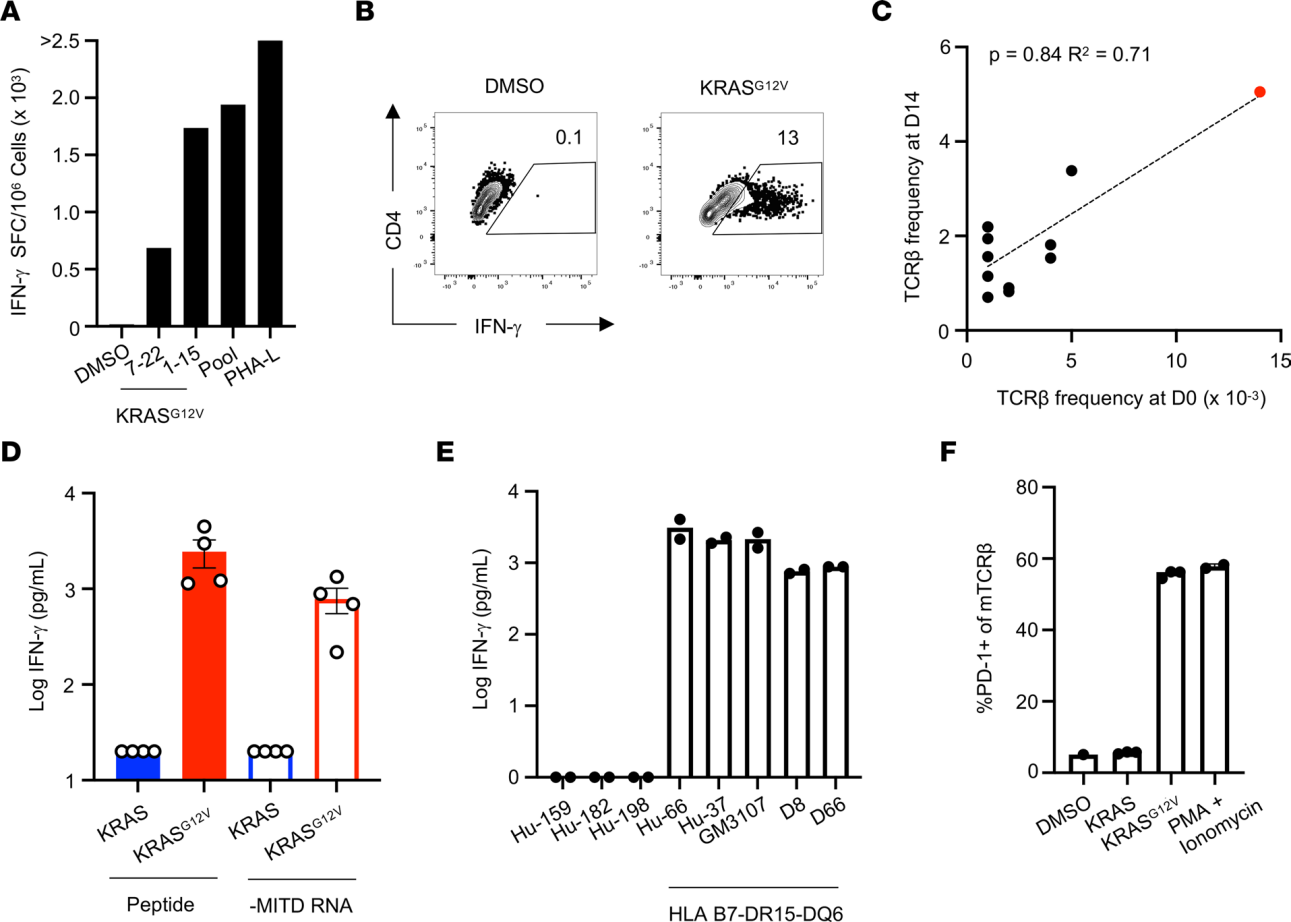
Identification of an HLA-DRB5*01:01–restricted, KRAS^G12V^-specific TCR from blood of a patient with pancreatic ductal adenocarcinoma. (**A**) IFN-γ ELISPOT assay results from ex vivo–expanded PBMCs restimulated with 5 μg/mL of the indicated peptides or pools. Data represent the average of 3 technical replicates in a single experiment. SFC, spot-forming cells. (**B**) Cytokine secretion assay results from ex vivo–expanded PBMCs restimulated with 5 μg/mL of the indicated peptides. (**C**) Spearman’s correlation of TCRβ frequencies among PBMCs before and after ex vivo peptide expansion. (**D**) IFN-γ secretion by TCR-engineered primary CD4^+^ T cells stimulated with autologous B-LCLs pulsed or electroporated with 1 μg/mL of the indicated peptides or in vitro–transcribed RNA, respectively. Data are representative of 4 independent experiments. (**E**) IFN-γ secretion by TCR-engineered primary CD4^+^ T cells stimulated with indicated B-LCLs pulsed with 1 μg/mL KRAS^G12V^ peptide. Data are representative of 2 independent experiments. (**F**) PD-1 upregulation by TCR-engineered primary CD4^+^ T cells stimulated with a DRB5*01:01–transfected murine fibroblast cell line pulsed with 1 μg/mL of the indicated peptides. Data are representative of 2 independent experiments.

Following these results, we set out to determine whether the inability of tumor cells to present antigen on MHC-II was true for other oncogenes. Given that circulating tumor-specific T cells have been identified in human patients with cancer (8, 22), we stimulated PBMCs from a patient (Hu-66) with pancreatic ductal adenocarcinoma (Supplemental Table 1) with a pool of peptides corresponding to common oncogenic mutations (Supplemental Table 6) for 14 days before restimulation with individual peptides to identify relevant T cell responses by ELISPOT. These results indicated the presence of a T cell response directed against KRAS^G12V^, as evidenced by an increased number of IFN-γ spots over background in response to KRAS^G12V^ amino acids 1–15 (Figure 5A). To identify the relevant TCRs associated with this response, we again used the cytokine secretion assay to sort IFN-γ–secreting cells. The cytokine secretion assay revealed specific production of IFN-γ by CD4^+^ T cells in response to restimulation with KRAS^G12V^ (Figure 5B). Comparing the TCRβ chains from sorted IFN-γ–secreting cells with those present in unexpanded PBMCs from this patient revealed a single TCR clonotype present at the highest frequency among both populations (Figure 5C and Supplemental Table 2). These results indicate that the frequency of this T cell clone was expanded at baseline in PBMCs, suggestive of a naturally arising in vivo response rather than in vitro priming in our expansion culture.

To verify the antigen specificity of this TCR, we expressed it in primary human CD4^+^ T cells by retroviral transduction and cultured these cells with autologous B-LCLs pulsed with either the mutant KRAS^G12V^ or corresponding WT peptide. Engineered T cells secreted IFN-γ when stimulated with the mutant, but not WT, KRAS peptide, verifying that this TCR is specific for KRAS^G12V^ (Figure 5D). Consistent with our results from the E6 TCR, engineered T cells expressing the KRAS^G12V^-specific TCR were also able to recognize B-LCLs expressing a fragment of KRAS^G12V^, but not WT KRAS, directed to the endosome by inclusion of an MITD, suggesting that this TCR recognizes endogenously processed and presented antigen (Figure 5D).

To identify the restricting HLA allele, we repeated these peptide stimulation experiments using B-LCLs expressing different combinations of HLA genes. B-LCLs expressing the common HLA-B7-DR15-DQ6 haplotype could stimulate the engineered T cells, whereas B-LCLs without these alleles could not (Figure 5E). Ultimately, stimulation of these cells by peptide-pulsed IHW03304, a mouse DAP3 cell line transfected with human HLA-DRB5*01:01, verified this as the restricting HLA molecule (Figure 5F).

### MHC-II^+^ NCI-H2444 and DAN-G tumor cells do not present KRAS^G12V^-derived epitope to CD4^+^ T cells.

**Figure 6:**
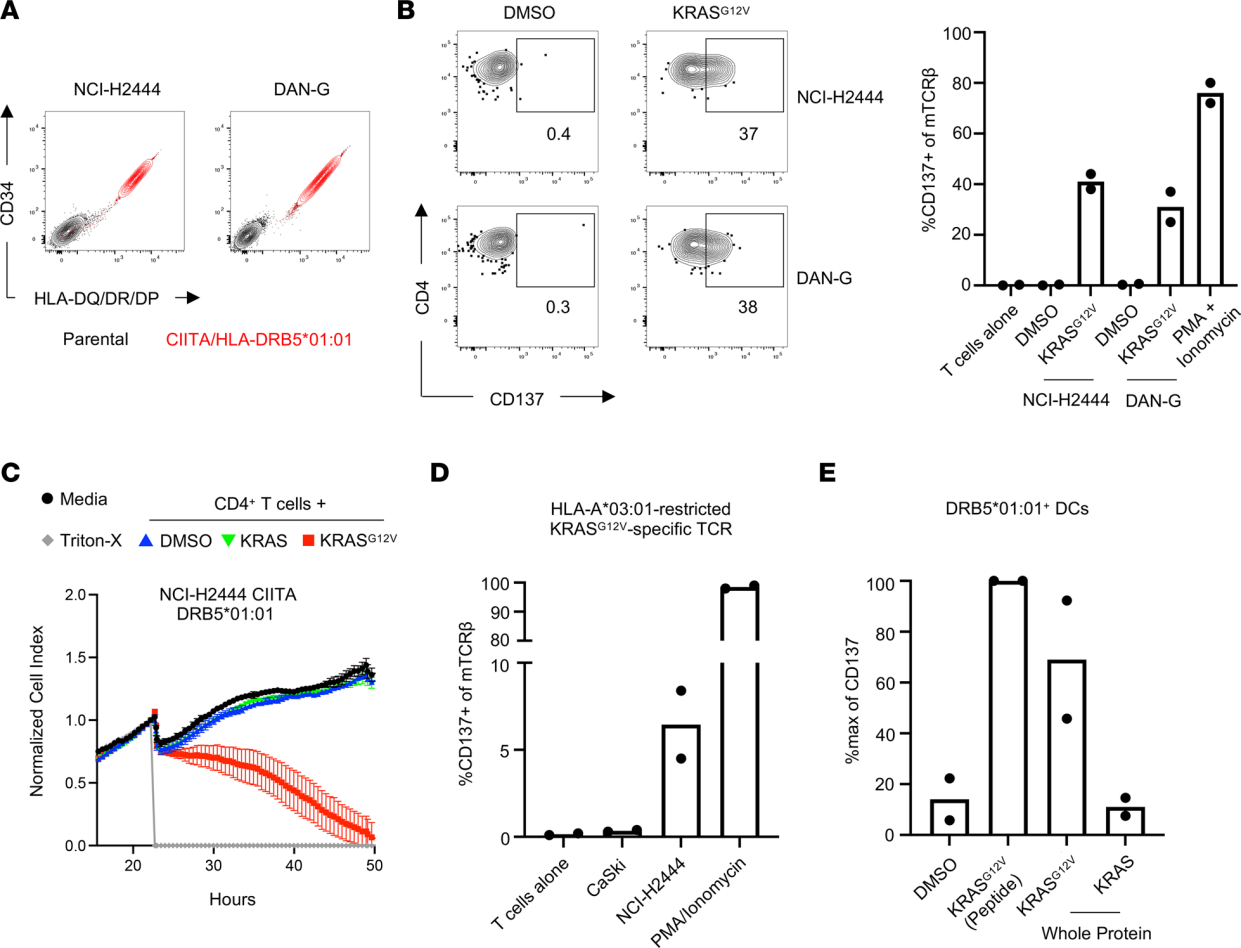
MHC-II^+^ KRAS^G12V+^ tumor cells do not present KRAS^G12V^-derived epitopes to CD4^+^ T cells. (**A**) Expression of CD34 reporter gene and HLA-DQ/DR/DP by indicated human tumor cell lines transduced with HLA-DRB5*01:01 and CIITA. (**B**) Representative FACS plots (left) and quantification (right) of CD137 upregulation after coculture of TCR-engineered CD4^+^ T cells with designated CIITA/HLA-DRB5*01:01–expressing tumor lines with or without 1 μg/mL KRAS^G12V^ peptide. Data represent 2 independent experiments. (**C**) Cellular impedance measurements to determine the kinetics of tumor cell death after recognition of NCI-H2444 CIITA tumor cell lines by KRAS^G12V^-specific, TCR-engineered CD4^+^ T cells compared with cells pulsed with 1 μg/mL KRAS^G12V^ or WT KRAS peptide. Normalized cell index values over time for the effector-to-target (E:T) ratio 10:1 is shown compared with tumor cells cultured without effectors. Data are presented as mean values of 3 technical replicates ± SEM and are representative of 2 independent experiments. (**D**) Quantification of CD137 upregulation by TCR-engineered CD8^+^ T cells expressing an HLA-A*03:01–restricted TCR specific for KRAS^G12V^ cocultured with CaSki or NCI-H2444 tumor cells. Data represented as the mean of 2 independent experimental replicates. (**E**) Quantification of CD137 upregulation by TCR-engineered CD4^+^ T cells cocultured with HLA-matched DCs pulsed with 1 μg/mL KRAS^G12V^ peptide or fed 20 μg/mL KRAS^G12V^ or KRAS whole protein overnight. CD137 upregulation was normalized to the peptide-pulsed control sample. Data are presented as the mean of 2 independent experimental replicates.

Next, we investigated the capacity of tumor cells to directly present KRAS^G12V^-derived epitopes on MHC-II. To generate HLA-matched MHC-II^+^ cell lines for these studies, we transduced the KRAS^G12V+^ cell lines NCI-H2444 and DAN-G, derived from human lung and pancreatic cancers, respectively, with CIITA and a construct encoding HLA-DRB5*01:01 with a truncated CD34 reporter gene (Figure 6A). Consistent with our previous results, MHC-II^+^ tumor cells expressing the restricting HLA allele were unable to stimulate TCR-engineered T cells unless target cells were first pulsed with exogenous peptide (Figure 6B). Importantly, there were no listed somatic mutations in MHC-II presentation genes (namely, *HLADMA*, *HLADMB*, *CD74*) for either NCI-H2444 or DAN-G in publicly available sequencing data (23). NCI-H2444 CIITA cells grew uninhibited in the presence of TCR-engineered T cells, whereas NCI-H2444 CIITA cells pulsed with KRAS^G12V^ peptide experienced delayed tumor growth (Figure 6C). Consistent with the results obtained for HNSCC-56, CD8^+^ T cells expressing an HLA-A*03:01–restricted, KRAS^G12V^-specific TCR (24) were able to recognize NCI-H2444 cells but not CaSki cells, which express HLA-A*03:01 but not KRAS^G12V^ (Figure 6D). These results demonstrate that a distinct oncogene present in the cytosol also cannot be presented by MHC-II^+^ tumor cells despite being effectively presented on MHC-I.

Expression of coinhibitory ligands such as programmed cell death ligand 1 (PD-L1) by tumor cells limits TCR signaling in T cells (25). To determine whether this mechanism may be preventing recognition of MHC-II^+^ tumor cells by CD4^+^ T cells, we cultured CD4^+^ T cells expressing our KRAS^G12V^-specific TCR or the E6-specific F12 TCR with NCI-H2444 CIITA and HNSCC-56 CIITA, respectively, with or without anti–PD-L1–blocking Abs. Blocking of PD-L1/PD-1 interactions was not sufficient to promote tumor cell recognition (Supplemental Figure 3). Similarly, addition of anti–CD28 agonist Ab did not modulate tumor cell recognition. These results suggest that a lack of antigen presentation, rather than the presence or absence of coinhibitory or costimulatory signals, respectively, is responsible for this phenotype.

Some studies suggest that endogenous proteins internalized from the plasma membrane are preferentially loaded onto MHC-II for presentation, compared with other subcellular compartments (26). Although KRAS proteins are frequently associated with membranes via lipid modifications, they do not express a transmembrane domain, and these interactions are not stable (3, 27). We reasoned, therefore, that anchoring the immunogenic fragment of KRAS^G12V^ to the plasma membrane of tumor cells may enable direct antigen recognition by CD4^+^ T cells. However, tumor cells expressing the KRAS^G12V^-MITD construct, as evidenced by expression of a P2A-linked EGFP reporter, were similarly unable to stimulate TCR-engineered CD4^+^ T cells (Supplemental Figure 4). These results suggest that subcellular oncogene localization is not the sole determinant of direct presentation on MHC-II by tumor cells. In addition, these results suggest that overexpression of the antigen is not sufficient to promote presentation on MHC-II.

Our results thus far suggest that tumor-specific CD4^+^ T cells may be more likely to recognize antigen presented by APCs than by tumor cells themselves. Therefore, we assessed the capacity of HLA-DRB5*01:01^+^ DCs to present antigen derived from exogenous protein. HLA-matched DCs generated in vitro were capable of stimulating TCR-engineered CD4^+^ T cells expressing the KRAS^G12V^-specific TCR when immature DCs were either pulsed with the KRAS^G12V^ 20-mer peptide or fed KRAS^G12V^ whole protein but not KRAS WT protein (Figure 6E). These results suggest that although the TCR cannot recognize endogenous antigen presented by tumor cells, it can recognize crosspresented exogenous antigen in the context of DCs.

## Discussion

The goal of this study was to determine whether TCRs isolated from naturally primed T cells obtained from patients with cancer could recognize tumor cells expressing oncogenes. With respect to the class I (HLA-A*02:01)–restricted, HPV-16 E6_29-38_ TCR obtained from HNSCC TILs, recognition was, perhaps unsurprisingly, affirmed for both an autologous tumor cell line and a well-characterized HLA-matched and E6-expressing tumor cell, CaSki (importantly, see Figure 2). The same TILs (Figure 1) also contained significant numbers of IFN-γ–producing E6-specific CD4^+^ T cells (Figure 3), which, in contrast, did not recognize E6-expressing tumor cells, even when they were induced to express ample surface MHC class II of the correct presenting allele (DQA1*01:02/DQB1*05:02) by IFN-γ treatment or CIITA transduction (Figure 4). CIITA expression induces not only the surface expression of MHC-II proteins but also HLA-DM and invariant chain molecules, which are required for antigen processing and presentation (28). HLA-matched B cells, in contrast, were recognized by this TCR when the antigen was directed to the MHC-II pathway. A similar situation was observed in the case of a KRAS^G12V^-specific, HLA-DRB5*01:01–restricted TCR, which could recognize HLA-matched B cells, but not MHC-II–expressing tumor cells, when antigen was directed to the MHC-II presentation pathway. Both TCRs possessed sufficient functional avidity to mediate recognition of endogenously expressed antigen under the conditions described above, and both could mediate cytotoxicity of peptide-loaded target cells. This latter observation mirrors the circumstance presented in several reports on cytotoxic CD4^+^ T cells, although our data extend the context of this observation by showing that endogenous expression of the source antigen and induction of MHC-II by IFN-γ is unlikely to yield a physiologic target on epithelially derived tumor cells for TCRs against nuclear or membrane-associated antigens.

Recent studies of human bladder cancer and melanoma identified genetic signatures corresponding to cytotoxic CD4^+^ T cells among TILs (14, 15). Functionally, these cytotoxic CD4^+^ TILs are capable of direct MHC-II–dependent tumor recognition, ultimately leading to target lysis by granzymes in a manner similar to their CD8^+^ counterparts. However, therapeutic translation of these findings may be limited by the fact that few solid tumors express MHC-II. Indeed, results of 2 independent studies suggested that only approximately one-third of patients with melanoma have any MHC-II^+^ tumor cells, and the frequency of these cells within the tumor is often less than 10% (15, 29). Instead, the predominant cellular source of MHC-II in the tumor microenvironment appears to be infiltrating leukocytes (26).

Although some solid-tumor cells do express either constitutive or inducible MHC-II, our results indicate that despite the presence of oncogene-specific CD4^+^ T cells among TILs and PBMCs, these cells cannot directly recognize and lyse antigen-expressing MHC-II^+^ tumor cells, whereas epitopes from the same protein may be effectively presented to CD8^+^ T cells on MHC-I (13). This suggests that simply expressing a target antigen may not be sufficient to confer recognition by CD4^+^ T cells for the subset of tumors that express MHC-II. Furthermore, these results appear to be consistent across multiple tumor types, because the cell lines queried in this study include those derived from HNSCC, lung adenocarcinoma, and pancreatic cancer.

Direct CD4^+^ T cell recognition of melanoma cell lines expressing MHC-II naturally or after CIITA transduction has been reported in 2 studies (30, 31). However, in both of these studies, a significant fraction of the TCRs tested did not recognize tumor cells directly. It is unclear whether these TCRs represent “bystander” clonotypes that do not recognize tumor antigens or tumor-specific TCRs that are unable to recognize their cognate antigen due to antigen presentation deficiencies such as those highlighted in this study. Oliveira et al. (31) demonstrated that direct recognition by CD4^+^ T cells was restricted to 2 melanomas with extremely high tumor mutational burdens, suggesting that this phenotype may confer additional necessary alterations permitting MHC-II presentation of tumor antigens.

Although attaching an MITD to the immunogenic fragment of KRAS_G12V_ did not result in direct recognition of tumor cells by CD4^+^ T cells, studies have described a bias for endogenous peptides derived from membrane-bound proteins eluted from MHC-II, presumably due to membrane recycling (26, 32). The melanoma antigen Trp1 exists in the plasma membrane, which may facilitate direct presentation to CD4^+^ T cells by B16 tumor cells (33). Another melanoma-associated antigen, gp100, is presented by tumor cells to CD4^+^ T cells in a manner dependent on gp100’s transmembrane domain (34). In recent work investigating the role of neoantigen-specific CD4^+^ T cells in a murine sarcoma model, epitopes derived from a mutated integrin subunit, also a membrane-bound protein, were eluted from MHC-II on CIITA-transduced tumor cells (35). Our results suggest that although membrane-bound proteins may be preferentially trafficked to endosomes for direct presentation on MHC-II, the presence of an immunogenic membrane protein alone is not sufficient to promote this presentation.

Other nonclassical presentation pathways have been described that may allow cytosolic or nuclear proteins access to MHC-II, including autophagy-associated presentation of intracellular proteins and transporter associated with antigen processing–dependent presentation to CD4^+^ T cells, whereby antigenic peptides are presumably transferred from MHC-I to MHC-II during membrane recycling (36, 37). Direct MHC-II–dependent recognition of autologous tumor cells by CD4^+^ T cells specific for E7 epitopes has been reported (38, 39); however, these studies both concerned E7 proteins expressed by less prevalent oncogenic HPV subtypes (namely, HPV-33 and HPV-59) presented by cervical cancer cells on HLA-DR molecules. It is possible, therefore, that HPV subtype, tumor histology, and restricting HLA alleles may also be relevant factors in the differential presentation of nuclear HPV antigens on MHC-II^+^ tumor cells.

Although our data suggest that direct tumor cytotoxicity is not a likely mechanism of E6- and KRAS_G12V_-specific CD4^+^ T cells, other antitumor mechanisms of CD4^+^ T cells have been described, and adoptive transfer of tumor-specific CD4^+^ T cells in patients with cancer has demonstrated antitumor activity (7, 40, 41). Therefore, MHC-II–restricted TCRs such as those identified in this study may be useful for ACT in human patients, given such TCRs’ targeting of epitopes derived from driver oncoproteins restricted to HLA haplotypes estimated at 1.27% and 16.10% of the US White population, respectively, for HLA-DQA1*01:02/DQB1*05:02 and HLA-DRB5*01:01 (42). Notably, CD4^+^ T cells aid in the priming of tumor-specific CD8^+^ T cells by licensing antigen-bearing DCs in a CD40L-dependent manner (43–45). We demonstrate that the KRAS^G12V^-specific TCR identified in this study does recognize crosspresented antigen in the context of DCs, suggesting these cells may be able to contribute to antitumor immunity via this function. CD4^+^ T cells may also provide local help for CD8^+^ T cells within tumors by secreting cytokines such as IL-2 and IFN-γ, supporting CD8^+^ T cell survival and recruitment to the tumor (46). In addition, CD4^+^ TILs may recognize antigen in the tumor microenvironment on myeloid cells such as macrophages. Indeed, preclinical studies have demonstrated that in the absence of MHC-II on tumor cells, adoptively transferred Trp1 CD4^+^ T cells still can exert antitumor immunity dependent on IFN-γ and correlate with increased cytotoxic activity of macrophages (47). However, this phenomenon appears to be dependent on tumor antigen secretion, which we would also speculate is minimal in the case of most oncogenes. Overall, our study highlights the selectivity of direct presentation of endogenous antigens by MHC-II^+^ tumor cells and demonstrates that neither CIITA expression nor the presence of a membrane-bound antigen is sufficient alone to promote direct recognition of tumor cells by CD4^+^ T cells. More studies are needed to characterize which antigens may be presented directly by MHC-II^+^ tumor cells under what conditions to fully understand the context-dependent roles of CD4^+^ T cells in cancer.

## Methods

### Cell culture.

TILs were harvested and cultured as previously described (48). Briefly, primary tumor tissue was dissected into 2–3 mm fragments, which were placed in a 24-well plate and cultured in RPMI-1640 supplemented with 10% human AB serum, penicillin-streptomycin, HEPES, gentamicin, and 6,000 IU/mL IL-2. Extravasated TILs were cultured by half-media replacements every 2–3 days. Primary human T cells from peripheral blood were cultured in 50:50 medium consisting of 50% AIM V plus 50% RPMI-1640 supplemented with 10% human AB serum, penicillin-streptomycin (100 U/mL each; Gibco, Thermo Fisher Scientific), 300 IU/mL IL-2, 5 ng/mL IL-7, and 5 ng/mL IL-15. Patient-derived tumor cell lines were generated and cultured as previously described (17). Briefly, primary tumor tissue was cut into fragments smaller than 2 mm and dissociated using a GentleMACS Octo Dissociator (Miltenyi Biotec). Tumor cells were resuspended in F-Media containing 5 μM Rho kinase inhibitor and plated over a bed of irradiated 3T3 fibroblasts (30 Gy). After initial culture, tumor cells were propagated in a 3:1 mix of irradiated 3T3-conditioned medium and F-Media containing 5 μM Rho kinase inhibitor. CaSki (ATCC), NCI-H2444 (ATCC), DAN-G (ATCC), J76 Jurkat (ATCC), 3T3-CD40L, and B-LCLs were maintained in RPMI medium supplemented with l-glutamine and HEPES (10 mM; Gibco, Thermo Fisher Scientific), 10% FBS, 1 mM sodium pyruvate (Gibco, Thermo Fisher Scientific), 1× MEM non-essential amino acids (Gibco, Thermo Fisher Scientific), and penicillin-streptomycin (100 U/mL each; Gibco). We maintained 293GP (ATCC) in DMEM medium supplement with 10% FBS and penicillin-streptomycin (100 U/mL each; Gibco, Thermo Fisher Scientific). IHW03304, GM3107, KAS011, D8, and D66 cells were gifts from Alessandro Sette (La Jolla Institute for Immunology).

### ELISPOT assays.

TILs or PBMCs were plated at 100,000 cells per well in ELISPOT plates coated with anti–IFN-γ Abs (1-D1K, Mabtech). Cells were restimulated with HPV-16 E6 and E7 PepMix (JPT) peptide pools, peptide pool representing selected oncogenic mutations, or indicated individual peptides at 5 μg/mL for pools or 10 μg/mL for peptides for 22 hours before developing ELISPOT plates according to the manufacturer’s instructions (Mabtech). For a positive control, 5 μg/mL phytohemagglutinin-L was used.

### Ex vivo expansion culture.

PBMCs were plated with peptide pool representing selected oncogenic mutations at 5 μg/mL. Fresh medium containing 10 IU/mL IL-2 was added on days 4, 7, and 10. On day 14, PBMCs were restimulated for use in either ELISPOT or cytokine secretion assays.

### TCR sequencing.

TILs or PBMCs were restimulated with 5 μg/mL HPV-16 E6 PepMix (JPT) or KRAS^G12V^ peptide, and IFN-γ–secreting cells were fluorescently tagged using the Human IFN-γ Secretion Assay kit according to manufacturer’s instructions (Miltenyi Biotec). Single IFN-γ^+^ cells were sorted into a 96-well plate using a FACSAria (BD Biosciences) and RNA-Seq was performed using an Illumina Miseq to identify paired TCRα and β chains. For identification of the E6-specific CD4^+^ TCR clone, IFN-γ–secreting cells were sorted as above and TCRα and TCRβ sequences were amplified by nested, multiplexed PCR, as previously described (49). PCR products were submitted for Sanger sequencing (ETON Biosciences). For TCRβ frequency analysis, PBMCs before and after 14-day ex vivo expansion with indicated peptides were sent to Adaptive Biotechnologies.

### HPV expression analysis.

RNA was isolated from Hu-56 primary tumor cells and tumor cell line and submitted for bulk RNA-Seq. Paired-end sequencing reads were mapped using BWA (v0.7.12) (50) to the HPV complete genome (GenBank accession no. NC_001526.2) and its corresponding transcriptome. The resulting sequence alignment map file was converted to a sorted binary alignment map and indexed, followed by counting mapped reads using SAMtools (v1.2) (50).

### Whole-exome sequencing.

Whole-exome sequencing and RNA-Seq were performed at the La Jolla Institute for Immunology Sequence Core using Agilent whole-exome capture and Illumina kits, respectively. FFPE biospecimens were distributed by the Moores Cancer Center to Tempus for next-generation sequencing along with matched reference DNA using peripheral blood specimens. Sequence reads from exome sequencing of the tumor and normal samples were aligned to the reference genome GRCh38 using SpeedSeq Align (v0.1.0) (51). Exome variants were identified using SpeedSeq Somatic, and variants were annotated using SNPeff (v4.3i) (52). The RNA-Seq and whole-exome sequencing data for this manuscript have been uploaded to NCBI BioProject, accession number PRJNA924789.

### T cell retroviral transduction.

TCR nucleotide sequences were synthesized and cloned in the MSGV1 retrovirus backbone using a BioXP 3200 (Codex). The sequence was codon optimized for expression in human cells. The human TCR constant regions were exchanged for mouse TCR constant regions with substitutions to enhance surface expression and promote preferential pairing (53, 54). Human PBMCs were isolated from buffy coats. CD4^+^ or CD8^+^ T cells were removed by magnetic selection, and the negative fraction containing all remaining lymphocytes was cultured in T cell medium with 50 ng/mL anti–CD3 Ab (OKT3) for 2 days. TCR retroviral supernatants were generated by cotransfection of 293GP cells with the MSGV1 vector containing the relevant TCR sequence and RD113 plasmid. Two days after transfection, the retroviral supernatants were harvested and either used fresh or frozen at –80°C. Transductions were performed on RetroNectin-coated plates (Takara), as previously described (5). Murine TCRβ constant region expression was assessed by flow cytometry to determine transduction efficiency. TCR-engineered T cells were used no sooner than day 7 after initial stimulation.

### T cell functional assays.

We cocultured 5 × 10^4^ TILs, transduced T cells, or J76 Jurkat cells with 1 × 10^5^ B-LCLs pulsed overnight with indicated concentrations of peptide or 5 × 10^3^ tumor cells seeded the previous day in 96-well U-bottom or flat-bottom plates, respectively. Where indicated, anti–CD28 agonist (clone CD28.2, BioLegend) or anti–PD-L1 blocking (clone 29E.2A3, BioLegend) Abs were added to T cell and tumor cell cocultures at 5 and 10 μg/mL, respectively. For cytokine and activation marker assays, 1× Cell Activation Cocktail (BioLegend) containing PMA and ionomycin was used as a positive control. For HLA blocking studies, 20 μg/mL of the following Abs were added to peptide pulsed B-LCLs at least 2 hours prior to addition of T cells: anti–HLA-DQ (Tü169, BioLegend), anti–HLA-DR (L243, BioLegend), or mouse IgG2a isotype control (MOPC-173, BioLegend). Where indicated, tumor cells were cultured for 72 hours with 10 ng/mL recombinant IFN-γ prior to addition of T cells. Supernatants were harvested after 18–24 hours, IFN-γ was measured by ELISA (Thermo Fisher Scientific), and cell pellets were stained for flow cytometry. Cytotoxicity assays were performed by coculture of T cells with tumor cells at indicated effector to target ratios. Briefly, 5 × 10^3^ tumor cells were seeded and cultured overnight before adding T cells at the indicated ratios. Target cell lysis was determined using the xCELLigence Real-Time Cell Analyzer (ACEA Biosciences), which assessed electrical impedance due to cell adherence every 30 minutes until the end of the experiment. The data were analyzed using the xCELLigence RTCA software package, and results were reported as cell index normalized to 1 at the time when T cells were added.

### MHC-II presentation assay.

Synthetic DNA constructs encoding the following were generated and cloned into MSGV1 using a BioXP 3200 (Codex): the N-terminal 25 amino acids of the human HLA-B gene, followed by the first 50 amino acids of HPV-16 E6 or 25 amino acids of KRAS^G12V^ fused to the C-terminal 55 amino acids of human HLA-B, a T2A self-cleaving element, and the coding sequence of EGFP. Viral supernatants were generated as described above, and autologous B-LCLs were transduced on RetroNectin-coated plates after overnight stimulation on a bed of irradiated (0.5 Gy) 3T3 cells expressing human CD40L (3T3-CD40L). Transduction efficiency was assessed by flow cytometric quantification of EGFP expression. Transduced B-LCLs were stimulated with 3T3-CD40L in the presence of 200 IU/mL recombinant human IL-4 (PeproTech) for 2 successive rounds of 2–3 days prior to coculture with autologous TILs.

### Generation of DCs for antigen presentation assays.

DCs were generated using the plastic adherence method. Frozen PBMCs were thawed and plated in DC medium (RPMI-1640 supplemented with l-glutamine, 5% heat-inactivated human AB serum, and 100 U/mL each penicillin-streptomycin) for 2 hours at 37°C. Nonadherent cells were removed by washing with warm PBS, and adherent cells were cultured in DC medium supplemented with 800 U/mL GM-CSF (Tonbo, Cytek Biosciences) and 500 U/mL IL-4 (PeproTech) for 6 days. We added 1 μg/mL KRAS^G12V^ peptide or 20 μg/mL KRAS^G12V^ or WT KRAS protein (Abcam) directly to immature DCs overnight. The next day, DCs were harvested and cocultured with TCR-engineered T cells at a 1:1 ratio overnight before assessing CD137 upregulation by flow cytometry.

### Retroviral transduction of tumor cells.

The coding sequence of human CIITA was synthesized (Integrated DNA Technologies) and cloned into MSGV1 by Gibson Assembly. The coding sequences of HLA-DRB5*01:01 α and β chains separated by a P2A sequence were synthesized (Integrated DNA Technologies) and cloned into a modified pHAGE lentiviral vector upstream of a T2A sequence followed by a truncated CD34 reporter sequence by Gibson Assembly. Retroviral supernatants were generated with MSGV1, as described above. Lentiviral supernatants were generated by cotransfection of 293T cells with pHAGE, tat, rev, gag/pol, and vsv-g plasmids. Viral supernatants were collected 24 hours later and concentrated by centrifugation in Amicon Ultra 5 tubes (MilliporeSigma). Tumor cells were plated and, 1 day later, the medium was replaced with retroviral or lentiviral supernatant supplemented with 10 μg/mL polybrene. Four hours later, virus-containing supernatant was aspirated and replaced with culture medium. MHC-II^+^ and CD34^+^ tumor cells were sorted using a FACS Fusion cell sorter (BD Biosciences).

### Flow cytometry.

Cells were labeled with fluorochrome-conjugated monoclonal Abs to the following antigens: anti–human CD3–PE (OKT3, Tonbo, Cytek Biosciences), CD4–PE–Cyanine7 (SK3, Tonbo, Cytek Biosciences), CD8–APC (Hit8a, Tonbo, Cytek Biosciences), CD34–Alexa Fluor 647 (581, BioLegend), CD69–PerCP–Cyanine5.5 (FN50, BioLegend), CD137–PE (4B4-1, Miltenyi Biotec), PD-1–BV650 (EH12.2H7, BioLegend), HLA-II–APC (Tü39, BioLegend), and anti–mouse TCRβ-APC (H57-597, Tonbo, Cytek Biosciences). Dead cells were stained with either DAPI or LIVE/DEAD Fixable Aqua (Invitrogen, Thermo Fisher Scientific). E6_29-38_–HLA-A*02:01–PE tetramer was assembled and labeled by the NIH Tetramer Core Facility. Cells were stained with 1 μg/mL tetramer for 1 hour on ice. Ab staining for surface antigens was performed for 15 minutes on ice. For intracellular cytokine staining (ICS), T cells were cocultured with peptide-pulsed APCs for 4–6 hours in the presence of Brefeldin A. Cells were permeabilized and stained for intracellular cytokines with the Cytofix/Cytoperm Kit (BD Biosciences) after surface staining, according to manufacturer’s instructions. The following fluorochrome-conjugated Abs were used for cytokine staining: IFN-γ–APC (4S.B3, BioLegend), IL-2–FITC (MQ1-17H12, BioLegend), TNF-α–BV785 (Mab11, BioLegend), and Granzyme B–PE (QA16A02, BioLegend). Data were acquired with a FACSCelesta flow cytometer (BD Biosciences) and analyzed with FlowJo v10.7 software.

### Statistics.

Statistical analyses were performed with Prism 8 (GraphPad Software). For ICS comparisons, an unpaired 2-tailed *t* test was used. For comparing HLA blocking Ab–mediated suppression of T cell signaling, a 2-way ANOVA was used. *P* < 0.05 was considered statistically significant.

### Study approval.

The deidentified human samples used in this study were obtained under informed consent through UCSD IRB protocol 101391CX, “Tumor Environment Phenotyping and Cell Isolation.” Written informed consent was provided by the individuals in the study.

## Author contributions

SEB, MSN, and SPS designed the experiments. SEB, MSN, RRT, AB, LM, and MB performed experiments. ALRP and JAG performed bioinformatic analyses under supervision of BP. SEB and MSN analyzed data and performed statistical analyses. EEWC and AMM provided clinical samples and data. SEB and MSN prepared the figures and wrote the manuscript with SPS. Order of co–first authors was determined by an objective assessment of the relative amount of the work attributable to each author by the last author.

## Supplementary Material

Supplemental data

Supplemental table 5

## Version 1 12/13/2022

In-Press Preview

## Version 2 01/24/2023

Electronic publication

## Version 3 01/24/2023

The Acknowlegdments section was updated to attribute the Graphical Abstract to Biorender.com
