# Supplementary material for: Tumor cells fail to present MHC-II–restricted epitopes derived from oncogenes to CD4+ T cells
Source: JCI Insight. 2023 Jan 24;8(2):e165570. doi: 10.1172/jci.insight.165570 (PMC9977289; doi:10.1172/jci.insight.165570)
Supplement: Supplemental data [file jciinsight-8-165570-s215.pdf]

Supplemental Table S1: Patient details.

ID	Age	Sex	Diagnosis	Treatment History	Mutations
Hu-56	58	F	Right base of tongue squamous cell carcinoma (P16+)	Transoral robotic resection/neck dissection (pT1N2bcM0) and adjuvant chemoradiation	unknown
Hu-66	60	M	Pancreatic head adenocarcinoma	Neoadjuvant Gemcitabine/Abraxane followed by pancreaticoduodenectomy (pT3N1cM0) and adjuvant Capecitabine/Gemcitabine	KRAS G12V, CDKN2A/B loss of exons 2-3, TP53 G244C

Supplemental Table S2: TCR gene sequences.

TCR	Coreceptor	V	J	CDR3α	CDR3β
F29	CD8	TRAV14/DV4*02, TRBV7-6*01	TRAJ58*01, TRBJ1-5*01	CAMREPRATSGSRLTF	CASSLGEGFSNQPQHF
F12	CD4	TRAV9-2*01, TRBV6-1*01	TRAJ40*01, TRBJ1-2*01	CALSHYSGTYKYIF	CASSEGTDYGYTF
KRAS	CD4	TRAV13-2, TRBV5-4	TRAJ53, TRBJ1-1	CAENSGGSNYKLTF	CASSWERAGKAFF

Supplemental Table S3: Individual peptides contained within E6 pepmix.

Peptide ID	AA Sequence
E6-1	MHQKRTAMFQDPQER
E6-2	RTAMFQDPQERPRKL
E6-3	FQDPQERPRKLPQLC
E6-4	QERPRKLPQLCTELQ
E6-5	RKLPQLCTELQTTIH
E6-6	QLCTELQTTIHDIIL
E6-7	ELQTTIHDIILECVY
E6-8	TIHDIILECVYCKQQ
E6-9	IILECVYCKQQLLRR
E6-10	CVYCKQQLLRREVYD
E6-11	KQQLLRREVYDFAFR
E6-12	LRREVYDFAFRDLCI
E6-13	VYDFAFRDLCIVYRD
E6-14	AFRDLCIVYRDGNPY
E6-15	LCIVYRDGNPYAVCD
E6-16	YRDGNPYAVCDKCLK
E6-17	NPYAVCDKCLKFYSK
E6-18	VCDKCLKFYSKISEY
E6-19	CLKFYSKISEYRHYC
E6-20	YSKISEYRHYCYSLY
E6-21	SEYRHYCYSLYGTTL
E6-22	HYCYSLYGTTLEQQY
E6-23	SLYGTTLEQQYNKPL
E6-24	TTLEQQYNKPLCDLL
E6-25	QQYNKPLCDLLIRCI
E6-26	KPLCDLLIRCINCQK
E6-27	DLLIRCINCQKPLCP
E6-28	RCINCQKPLCPEEKQ
E6-29	CQKPLCPEEKQRHLD
E6-30	LCPEEKQRHLDKKQR
E6-31	EKQRHLDKKQRFHNI
E6-32	HLDKKQRFHNIRGRW
E6-33	KQRFHNIRGRWTGRC
E6-34	HNIRGRWTGRCMSCC
E6-35	GRWTGRCMSCCRSSR
E6-36	GRCMSCCRSSRTRRE
E6-37	SCCRSSRTRRETQL

Supplemental Table S4: HLA class II genotyping of B cell lines.

B Cell Line	DQA1	DQA2	DQB1	DQB2	DRB1	DRB2	DRB3	DRB4	DRB5
Hu-56	01:02	05:05	03:01	05:02	11:01	16:01			
Hu-195	01:04	05:05	03:01	06:01	13:03	14:04			
KAS011	01:02	01:02	05:02	05:02	16:01	16:01			
Hu-66	01:02	05:01	02:01	06:02	03:01	15:01	01:01		01:01
Hu-37	01:02	03:03	02:02	06:02	04:03	15:01		01:03	01:01
Hu-159	05:05	02:02	03:19	07:01	11:02	15:01	03:01	01:01	
Hu-198	01:03	05:01	02:01	06:03	03:01	13:01			
D66	N/A	N/A	05:01	06:02	01:01	15:01			01:01
D8	01:02	01:03	06:02	06:03	13:01	15:01			01:01
GM3107	N/A	N/A	06:02	06:02	15:01	15:01			01:01

Supplemental Table S6: Peptides representing recurrent cancer mutations.

Peptide Sequence	Gene	aaPos	aaRef	aaAlt	mut.pos
FGLATEKSRWSGSHQFEQLS	BRAF	600	V	E	6
EDLTVKIGDFGLATEKSRWS	BRAF	600	V	E	15
VVVGADGVGKSALTIQLIQN	KRAS	12	G	D	6
MTEYKLVVVGADGVGKSALT	KRAS	12	G	D	12
VVVGAVGVGKSALTIQLIQN	KRAS	12	G	V	6
MTEYKLVVVGAVGVGKSALT	KRAS	12	G	V	12
LSEITKQEKDFLWSHRHYCV	PIK3CA	545	E	K	6
LKAISTRDPLSEITKQEKDF	PIK3CA	545	E	K	15
PSGGDHFCLGQLSNVHRTEA	SMAD4	361	R	H	6
IVTVDGYVDPSGGDHFCLGQ	SMAD4	361	R	H	15

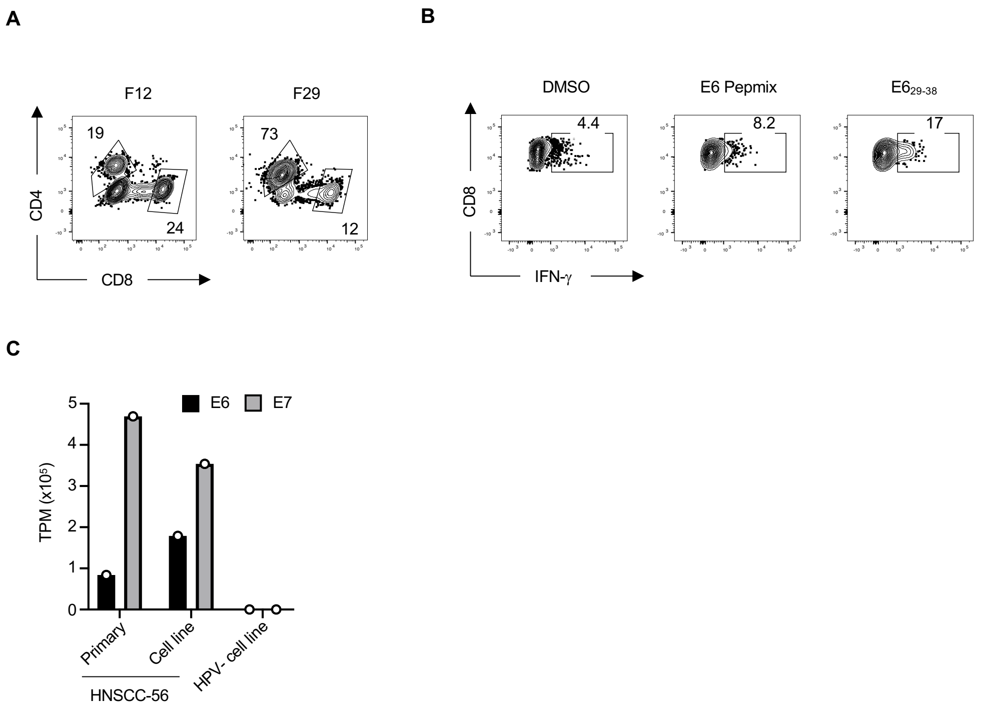

Supplemental Figure S1: Cellular composition and epitope specificity of F29 TIL and HPV RNA expression by Hu-56 cell line and primary tumor. A Representative flow cytometry plots demonstrating percentage of $CD4^+$ and $CD8^+$ TIL among F12 and F29. **B** Cytokine secretion assay results demonstrating secretion of IFN-γ by $CD8^+$ TIL after stimulation with either E6 pepmix or $E6_{29\text{-}38}$. Representative FACS plots from two independent experiments. **C** Quantification of RNA transcript reads expressed as transcripts per million (TPM) mapping to the indicated HPV-16 genes in HNSCC-56 primary tumor, HNSCC-56 cell line, and an HPV^- control cell line.

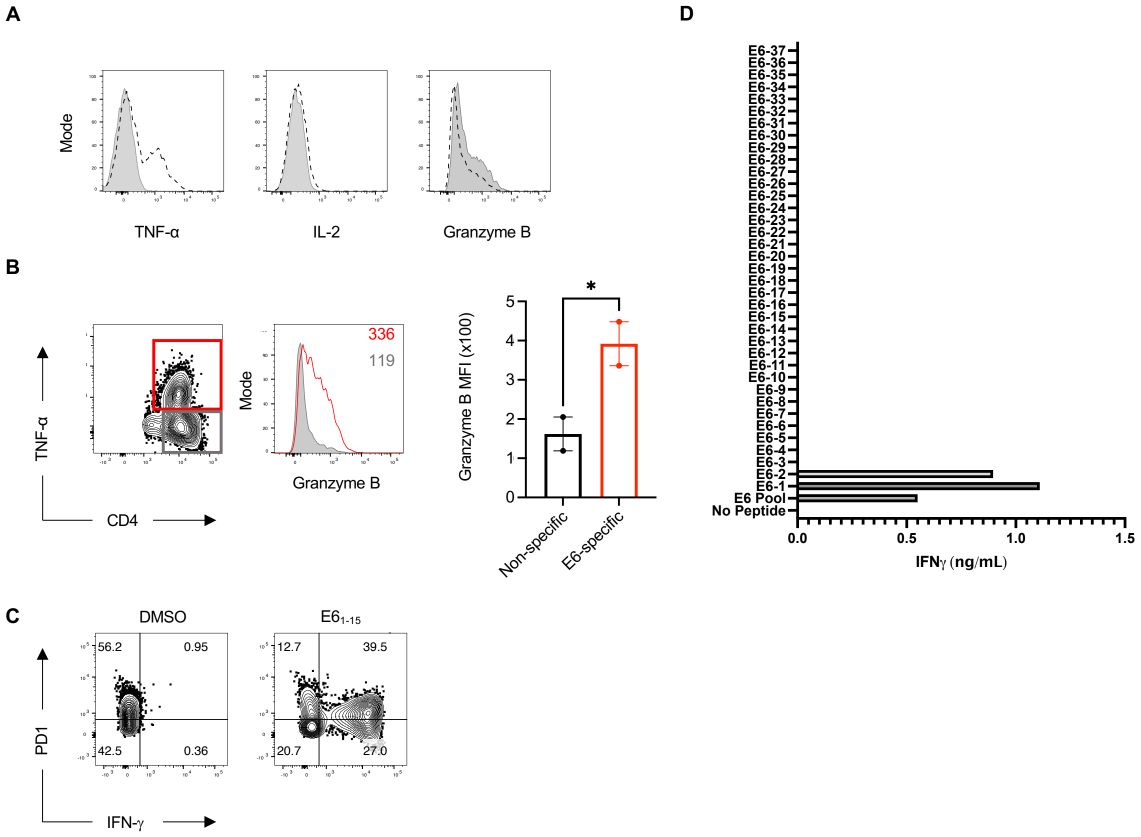

Supplemental Figure S2: Cytokine production, surface phenotype, and epitope specificity of E6-specific CD4⁺ TIL. A Representative FACS plots demonstrating intracellular cytokine staining of unstimulated (grey) or E6-stimulated (dashed line) F12 REP TIL for indicated cytokines. **B** TNF-α + and – cells were gated as a surrogate for E6-specific and non-specific, respectively, to determine the relative abundance of intracellular granzyme B in these two subsets. Data are representative of mean +/- SEM from 2 independent experiments. * $p<0.05$ paired T-test. **C** Representative FACS plots of unstimulated (left) or E6-stimuilated (right) F12 REP TIL stained for intracellular IFN-γ and surface PD1. **D** Individual peptides from the E6 pepmix (Table S1) were pulsed onto autologous B-LCLs prior to culture with E6-specific CD4⁺ TIL. IFN-γ secretion was measured by ELISA. Data is representative of 2 independent experiments.

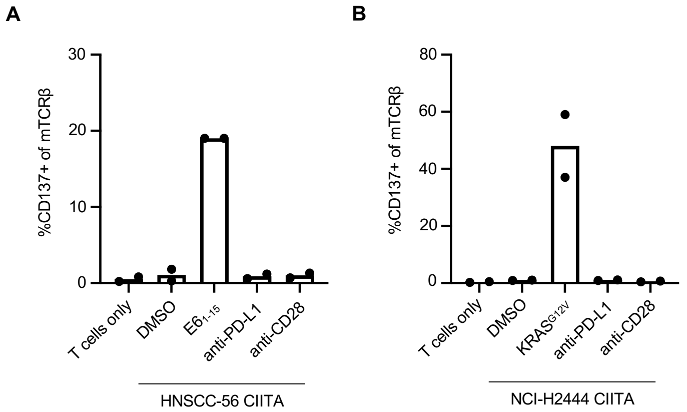

Supplemental Figure S3: Addition of anti-CD28 agonist or anti-PD-L1 blocking antibodies do not impact tumor recognition by $CD4^+$ T cells. A Quantification of CD137 upregulation after coculture of E6-specific F12 TCR-engineered $CD4^+$ T cells with HNSCC-56 CIITA tumor cells with or without 1 µg/ml $E6_{1-15}$ peptide, 10 µg/ml anti-PD-L1, or 5 µg/ml anti-CD28. Data represent two independent experiments. **B** Quantification of CD137 upregulation after coculture of $KRAS^{G12V}$-specific TCR-engineered $CD4^+$ T cells with NCI-H2444 CIITA tumor cells with or without 1 µg/ml $KRAS^{G12V}$ peptide, 10 µg/ml anti-PD-L1, or 5 µg/ml anti-CD28. Data represent two independent experiments.

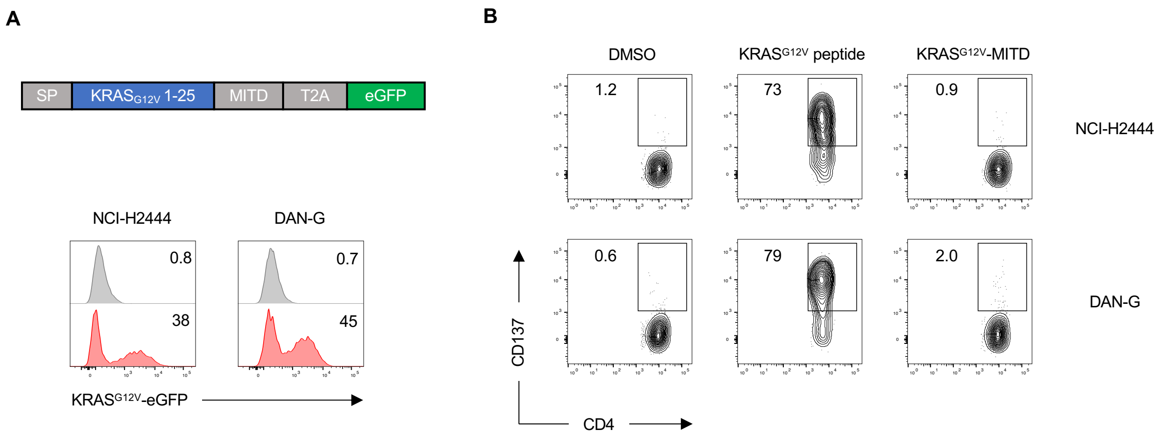

Supplemental Figure S4: Membrane-anchored KRAS^{G12V} is not sufficient to promote direct recognition by CD4^{+} T cells. A Schematic of KRAS^{G12V}-MITD construct (top) and transduction efficiency of NCI-H2444 and DAN-G measured by eGFP expression (bottom). **B** FACS plots demonstrating CD137 upregulation after coculture of TCR-engineered CD4^{+} T cells with designated CIITA/HLA-DRB5*01:01-expressing tumor lines pulsed with DMSO, KRAS^{G12V} peptide, or expressing the endogenous KRAS^{G12V}-MITD construct. Data representative of 2 independent experiments.
